# Conductive Polymer-Based Interactive Shelving System for Real-Time Inventory Management

**DOI:** 10.3390/s23218857

**Published:** 2023-10-31

**Authors:** Musafargani Sikkandhar, Ruiqi Lim, Ramona B. Damalerio, Wei Da Toh, Ming-Yuan Cheng

**Affiliations:** Institute of Microelectronics, A*STAR (Agency for Science, Technology and Research), Singapore 138634, Singapore

**Keywords:** smart-shelving, inventory management, piezoresistive, shape, weight sensor mat array

## Abstract

Stockouts constitute a major challenge in the retail industry. Stockouts are caused by errors related to manual stockkeeping and by the misplacement of items on shelves. Such errors account for up to 4% of lost sales. Real-time inventory management systems for misplaced items or missing stock detection in retail stores are limited. Accordingly, a conductive polymer-based interactive shelving system for real-time inventory management is developed. The system comprises an 80 × 48 sensor array fabricated by screen-printing a piezoresistive carbon-based conductive polymer layer onto gold interdigitated electrodes deposited on a flexible substrate. Each sensing pixel has dimensions of 5 mm × 5 mm and a sensing area of 4 mm × 4 mm. The sensor mat can detect the shape and weight features of stockkeeping units (SKUs), which can then be analyzed by a TensorFlow model for SKU identification. The developed system is characterized for functional resistance range, uniformity, repeatability, and durability. The accuracy of SKU identification achieved using shape features only and the accuracy of SKU identification achieved using both shape and weight features is 95% and 99.2%, respectively. The key novelty of the work is the development of a deep learning-embedded interactive smart shelving system for retail inventory management by using the shape and weight features of SKU. Also, the developed system helps to detect the SKU if they are stacked one over the other. Furthermore, multiple sensor mats implemented on various shelves in a retail store can be modularized and integrated for monitoring under the control of a single PC. Accordingly, the proposed retail inventory tracking system can facilitate the development of automated “humanless” shops.

## 1. Introduction

Inventory management is currently a challenging task in the retail industry owing to the exponential increase in the heterogeneity of both consumers and products. Specifically, the retail industry faces a considerable challenge in optimizing inventory record management and maintaining sales growth and low operational costs. Large-scale product lines in the retail industry engender difficulties in the tracking of sales profits, shelf life, and customer responses. Retailers generally perform stockkeeping manually and encounter mismatches in stocks due to misplacement of items on the wrong shelves. Such item misplacement typically accounts for 5–10% of all stockouts and up to 4% of lost sales [1]. Moreover, identifying misplaced items and then restocking such items on the appropriate shelves are time-consuming and costly tasks. A study conducted to gain a comprehensive understanding of the root cause of losses related to item misplacement revealed that such losses are primarily due to human error [2]. That is customers fail to place misplaced products on the appropriate shelves resulting in mismatch in records during manual stockkeeping.

The Internet of Things (IoT) has been extensively adopted in various aspects of daily life. Accordingly, an IoT system comprises a large network of sensors, connectors, actuators, and humans [3,4,5]. An IoT-enhanced inventory management system can help to improve operational efficiency, customer satisfaction, and reduce operational costs [6]. Moreover, an interactive inventory management system can enhance customers’ shopping experience and satisfaction. An inventory management system can use information obtained from multiple sources such as Electronic Product Code (EPC) scanners [7], radiofrequency identification (RFID) sensors [8], Global Positioning System (GPS) devices [9], and barcode readers to execute automatic stockkeeping unit (SKU) identification and tracking processes. Although EPC scanners are easy to implement for fast checkout, extensive manpower for the system implementation is required. On the other hand, RFID and GPS are low-cost technologies for retail stores implementation. These technologies are crucial for tasks such as stockkeeping, availability monitoring, theft detection, misplaced item detection, and fast check-out. Nevertheless, an inventory management system must be updated when new SKUs are added to a shelf, which may entail additional data entry tasks and increased labor costs. Moreover, the inventory management system involves large storage consumption costs due to tagging of RFID or GPS on each item. Also, the resolution of GPS is poor in indoor spacing. RF tagging or barcode are inefficient in detecting misplaced items or missing stock, as they needs a scanner system to identify the misplaced item.

Alternative systems have recently been developed, such as computer vision, which accurately identify the misplacement, misorientation, and theft incidents. Nevertheless, computer vision-based systems are associated with expensive system implementation [10]. Few researchers have also investigated using ultrasound sensors for retail management [11]. Due to its system complexity, there is limited usage for industrial application. The other low-cost sensors such as Infrared (IR) sensors are also limited in their market application due to their poor resolution and inaccuracy in SKU identification [12]. Systems based on pressure-sensitive sensors have several benefits, including sufficient resolution, minimal data-processing requirements, low manufacturing costs, and simple integration. Most pressure-sensitive sensors are capacitive devices. Nonetheless, such sensors are limited by problems related to pressure sensitivity and by the considerable hysteresis engendered by the viscoelastic characteristics of elastomers [13,14,15]. Compared with other types of sensors, piezoresistive conductive ink-based sensors exhibit superior uniformity, repeatability, linearity, and hysteresis. Moreover, they are cost-efficient and simple to manufacture [16]. Statistical tools are predominantly used for stock management, such as the Monte Carlo simulation method [17]. Also, the supply chain of the products plays a vital role in stock management. Hence, the optimal route path selection to reduce the cost and time is studied by Al-Tayar et. al., 2023 [18]. As large data are involved, cloud computing and storage is also implemented [19]. Other statistical tools such as Design of Experiments (DoE) have been employed for stock management [20].

Most studies have used piezoresistive conductive polymers to detect the shapes of objects placed on them. The conductive polymers are chosen due to their high sensitivity under ambient conditions, electrochemical properties, ease of varying the sensitivity through doping, mechanical resistivity, electro-mechanical characteristics, and good inherent charge transport characteristics [21]. Limin Gao et al. demonstrated that functionalization of traditional fibers with carbon nanotubes improves the sensitivity of acoustic detection [22]. Meanwhile, multiple researchers have demonstrated single [23] and multi-walled carbon nanotubes [24] and carbon nanoparticles [25] as conductive polymer for various piezo-resistive applications. In most of the studies, conductive polymer-based piezo resistive applications involve either shape identification through the applied pressure. However, the effective use of such polymers is challenging in the retail industry because operations in this industry include scenarios involving SKU misplacement, theft, stacking, and disorientation on shelves. Thus, using only the shape features of the SKUs is not efficient. Hence, detecting SKU weight and shape features using a piezoresistive sensor can facilitate inventory management in the retail industry. Conclusively, there is a need for a low-cost and easy to implement inventory tracking system that aids in detecting SKU misplacement and disorientation on shelves. The integration of machine learning algorithms and wireless communication enhances the system capability. Accordingly, the current study presents an interactive shelving system for real-time inventory management. The system comprises a smart 80 × 48 piezoresistive sensor array, which is fabricated by screen-printing a conductive polymer layer onto gold interdigitated electrodes deposited on a flexible substrate. Each sensing pixel has dimensions of 5 mm × 5 mm and a sensing area of 4 × 4 mm^2^. The sensor array provides information on the shape and weight features of SKUs, and this information is subsequently analyzed by a deep learning algorithm known as TensorFlow model [26] for SKU identification. Differentiating from the existing technologies, our proposed system is integrated with the TensorFlow model, which can help to identify the different SKUs that have been misplaced in an incorrect shelf. This model has already been trained with the information of shelves with corresponding SKUs. In addition, our system can identify the stacking of SKUs using the weight information. The system is characterized for functional resistance range, uniformity, repeatability, and durability. The key novelty of the work is the development of deep learning-embedded interactive smart shelving system for retail inventory management by using the shape and weight features of SKUs. Compared to the existing literature, the developed sensor mat is capable of detecting both the shape and weight features of the SKU. This enables efficiency in stockkeeping and reduction in misplacement. Also, the developed system helps to detect the SKUs if they are stacked one over the other. A comparison of different technologies used for real-time inventory management is shown in Table 1. In this table, we have compared the key performance indicators such as accuracy, estimated cost (USD), spatial resolution, and integration with artificial intelligence (AI). Hence, the developed interactive shelving system stands out against the existing technologies with respect to cost, accuracy, and spatial resolution.

## 2. Design Concept

**Figure 1:**
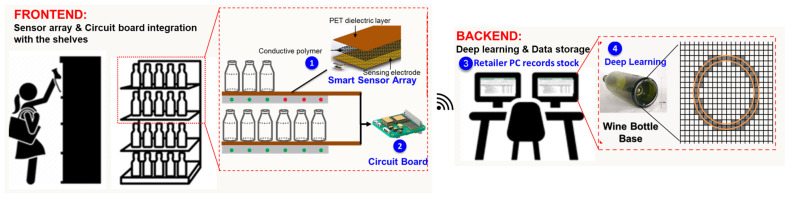
IoT-based smart shelving for retail inventory management.

Figure 1 illustrates a schematic of the design concept of the proposed real-time inventory management system. In this system, the sensor array detects the shape and weight of a SKU, and the detected information is then transmitted to a TensorFlow model—a deep learning algorithm—for SKU identification. Based on the identified SKUs, the system executes stock monitoring, and misplaced item detection. Because the system uses weight features for SKU identification, it can detect the stacking of identical SKUs. The circuit of the system can communicate wirelessly with a retail store’s PC; hence, the system can be used to monitor the movement of stock across the store. Accordingly, the system can thus improve the efficiency of stock management and reduce the labor requirements for retail stores.

**Figure 2:**
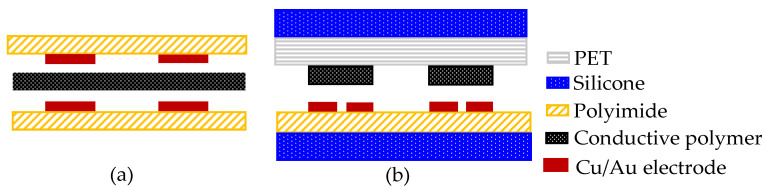
Sensor array stacking schematic: (**a**) conventional and (**b**) the proposed design.

The system comprises an array of sensors. A sensor array should ideally be thin and flexible, have an adequate resolution, and be devoid of crosstalk between its sensing pixels. However, most of the existing sensor mats based on conductive polymers cannot eliminate crosstalk between sensing elements because they are composed of a uniform (i.e., blanket) pressure-sensing layer; that is, neighboring sensing pixels are activated whenever the uniform conductive polymer is deformed [27,28]. To overcome this problem, a sensor array is designed that entails stacking conductive polymers on individual electrodes. Furthermore, instead of using conventional electrodes (Figure 2a), interdigitated electrodes are utilized (Figure 2b). The design can thus eliminate crosstalk between electrodes. Additionally, in contrast to conventional designs that involve stacking electrodes on top of one another, the design involves the implementation of a single layer of interdigitated electrodes on a polyimide substrate. Accordingly, the design improves on conventional designs by reducing the complexity of the electrical packaging of large sensor mats.

**Figure 3:**
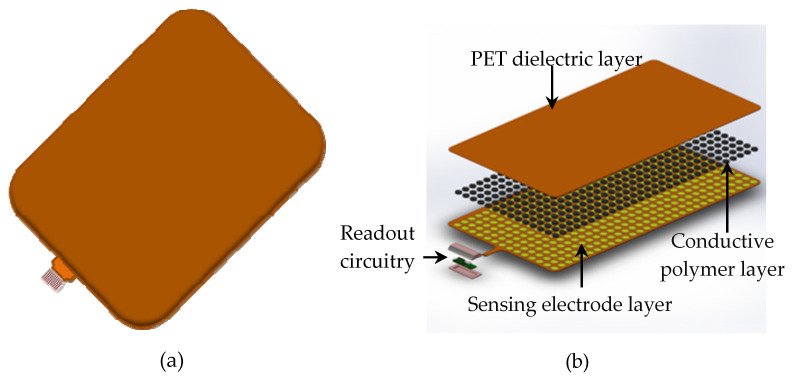
Schematic of the sensor mat: (**a**) assembled view and (**b**) exploded view.

The proposed sensor mat comprises three key layers: a sensing electrode layer, conductive polymer layer, and polyethylene terephthalate (PET) dielectric layers (Figure 3). It comprises a total of 3840 sensing pixels arranged in an 80 × 48 grid with electrical interconnections. Each sensing pixel has dimensions of 5 mm × 5 mm and a sensing area of 4 mm × 4 mm; the pixels were printed on gold interdigitated electrodes. When no force is applied to the sensor mat, the sensing pixels remain in an idle state, resulting in an open readout circuit and an infinite resistance value. However, when a force is applied to the mat, the conductive polymer layer between the sensing electrodes and PET dielectric undergoes a deformation, which triggers the conduction of current between the electrodes; thus, the resistance drop at each sensing pixel can be determined. In practice, resistance is correlated with SKU weight; hence, resistance can be used to detect the shape and weight of a SKU that is placed on the sensor mat.

## 3. Fabrication and Assembly

**Figure 4:**
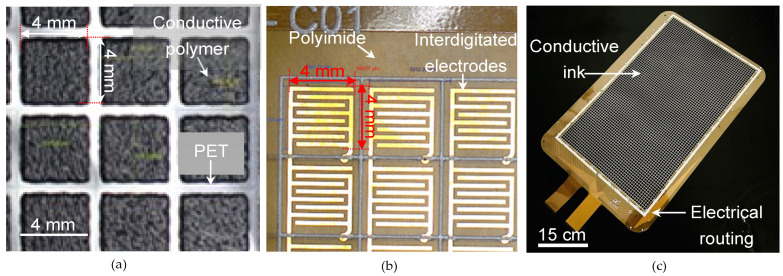
Images of the fabricated sensor mat layers: (**a**) array of conductive polymer on PET, (**b**) interdigitated sensing electrodes layer, and (**c**) assembled sensor mat.

The sensor mat was fabricated by screen-printing a piezoresistive conductive polymer layer (resistivity: 20 kΩ) onto a 100 μm-thick polyethylene terephthalate (PET) film measuring 60 cm × 36 cm. The conductive polymer used in the sensor array can operate at high temperatures (>150 °C) and can be screen-printed on PET. The conductive polymer required should have a thin form factor (<50 μm) and is sensitive to micro-Newton (μN) forces. Various conductive polymers are used to study the repeatability for screen printing, solubility, operating temperature for curing process, and thin form factor, as shown in Table 2. After evaluating various conductive polymer gels, AG125A (MOS Corporation Ltd., Hong Kong, China) is used as a carbon-based piezoresistive conductive polymer. The optimal thickness of this polymer is determined to be 6 μm by carefully evaluating the trade-off between sensitivity and degradation. The conductive polymer was screen-printed and cured at 110 °C for 10 min (Table 3). The thickness of the conductive polymer coating is validated by assessing the accuracy of its shape as detected on the graphical user interface (GUI). Images of the fabricated sensor mat layers are shown in Figure 4. After polymer curing, the array of interdigitated electrodes (gaps between electrodes: 250 μm) are packaged onto the conductive polymer layer (Figure 4a). These interdigitated electrodes (Figure 4b) were fabricated onto a layer of polyimide substrate through the standard electroless nickel immersion gold process, thus yielding a complete sensor array. The top and bottom of the fabricated sensor array were covered with a silicone mat with a thickness of 1.5 mm and Shore A hardness of 13 to concentrate the pressure of the SKUs onto the sensing pixels of the array. The silicone mat is used to enhance uniformity and reduce the risk of damage to the sensor array due to long-term loading. In the system, the sensor array is connected to a readout circuit board (comprising an analog-to-digital converter (ADC)), which translates the resistance drop in each sensing pixel to a heat spectrum on the GUI. The readout circuit operates at a sampling frequency of 10 fps and reads the arrays of sensing pixels row by row. Moreover, the GUI displays the shape and weight information of a SKU that is placed on the sensor mat.

## 4. Experimental Validation and Results

### 4.1. Benchtop Characterization of Sensor Mat

**Figure 5:**
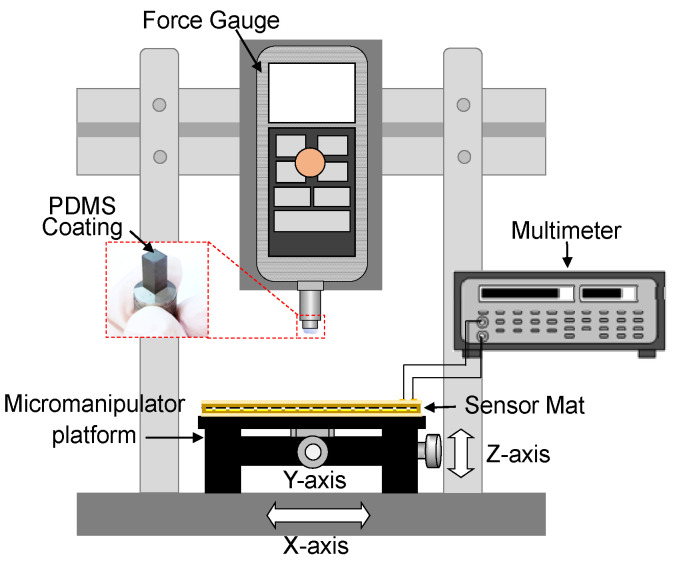
Experimental setup for sensor mat characterization.

To study the uniformity and repeatability of the sensor mat, a benchtop characterization is conducted by using a force gauge. The sensor mat is placed on the micromanipulator platform (*x*-*y*-*z*-axis stage) as shown in Figure 5. The sensor mat’s output pads are connected to a PCB adapter board with labels corresponding to the row (R) and column (C) of each sensing pixel. The output wires of rows and columns are connected to the digital multimeter. The force gauge is fixed on the holder with a gauge tip, which is coated with 2 mm-thick silicone substrate that was used to provide a reaction force. To test each sensing pixel, the sensor mat is moved in x–y–z direction so that the target sensing pixel touches the force gauge tip. When the sensing pixel touches the force gauge tip, force (in mN) is displayed by the force gauge and the corresponding resistance (KΩ) is read by the multimeter. The force associated with a resistance drop at nine different points is selected across the sensor mat. The uniformity of the sensor mat is validated by applying an incremental load (ranging from 10 to 75 g) to each of the nine points in a perpendicular direction to the flat stage of the force gauge; the collected data are then used to plot a graph.

**Figure 6:**
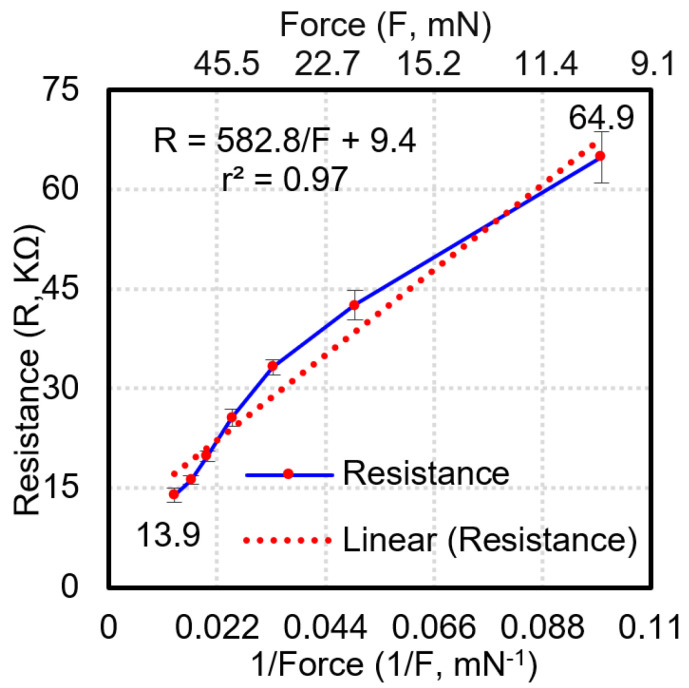
Experimental results of sensor mat characterization for uniformity.

**Figure 7:**
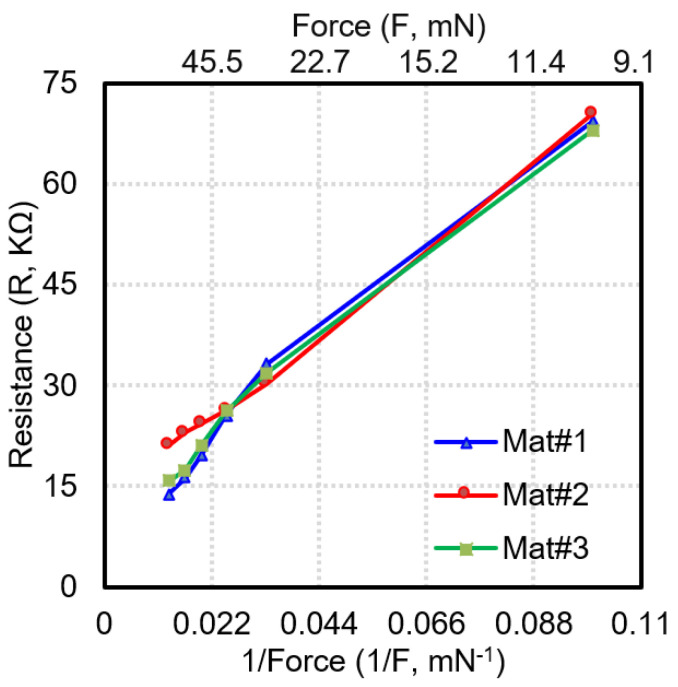
Experimental results of sensor mat characterization for repeatability.

The functional resistance range is between 13.9 and 64.9 kΩ as shown in Figure 6. The functional resistance range is the resistance measured at each pixel of the sensor mat when subjected to forces from 10 mN to 75 mN. The force range is determined based on the area of the sensing pixel of 5 × 5 mm^2^, which implies that if the force beyond 75 mN is exerted on the pixel, then the resistance measured at the sensing pixel is saturated. Hence, the minimum force resolution of each sensing pixel is 10 mN. On the basis of the graph, the following characterization formula is derived for resistance:

R = 582.8/F + 9.4

where R is the resistance of the sensing electrode and F is the applied load on the sensing electrode. The linearity of the fitting curve and coefficient of variation are 0.97 and 0.127, respectively. The repeatability of the sensor mat is assessed by repeating the same procedure on three different sensor mats and then plotting the resistance against the inverse of the force applied (Figure 7). The average standard deviation of the resistance for the uniformity and repeatability evaluation is calculated to be 1.6 KΩ and 2.29 KΩ, respectively, which is within 2 sigma deviation. The largest variation between the resistance values derived for the three sensor mats was observed at the lowest load (10 g).

### 4.2. Durability of Sensor Mat

**Figure 8:**
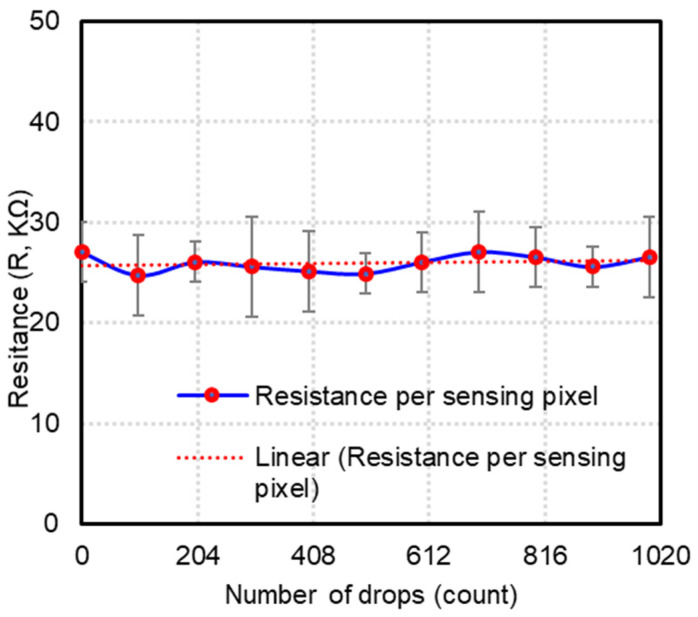
Experimental results of durability test of sensor mat.

The durability of the sensor arrays is studied by dropping a 1 kg standard weight on the sensor mat from a height of 10 cm 1000 times. This test was repeated at three different positions on the sensor mat. The resistance measured at the activated area was plotted against the number of drops to validate the durability of the sensor mat (Figure 8). The average resistance drop experienced by each sensing pixel after the completion of the test is approximately 25.9 kΩ. The average standard deviation for the derived resistance is 3.2 KΩ, and the highest standard deviation is approximately 5 KΩ.

### 4.3. Detection of SKU Shape and Weight

**Figure 9:**
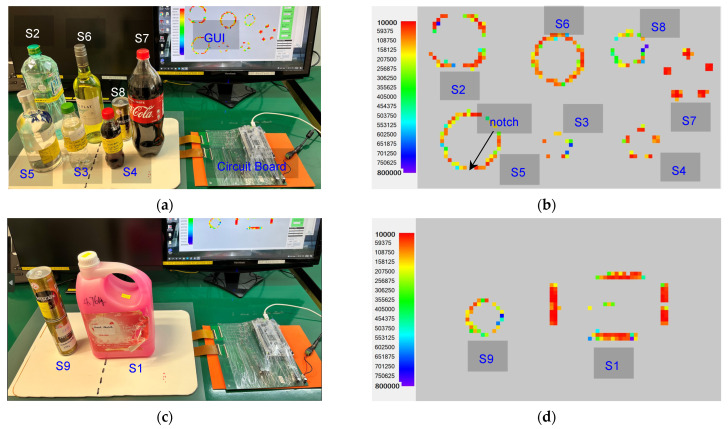
Images of SKU experimental setup on sensor mat and shape display heat map result: (**a**,**b**) SKU S2 to S8 placement and (**c**,**d**) stacking items and heavy-weight SKU (4.7 kg).

**Figure 10:**
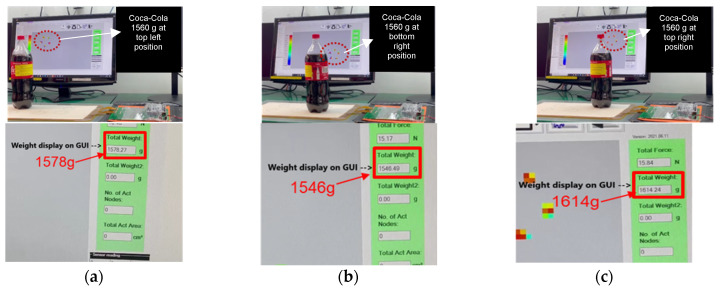
Images of S7 (Coca-Cola 1.5 L) experiment setup and weight display heat map of S7 at three different locations: the sensor mat (**a**) top left position, (**b**) bottom right position, and (**c**) top right position.

The sensor mat is connected to an external readout circuit board and GUI. The readout circuit board comprises an ADC unit, which translates analog resistance drop values into a heat map on the GUI. When a SKU is placed on the sensor mat, the intensity of the generated heat map depicts the shape of the SKU, and the weight information of the SKU is obtained by using the aforementioned characterization formula. The sensor mat’s shape and weight detection performance are evaluated by using nine SKUs as shown in Table 4. The nine different SKUs (S1–S9) are placed on the sensor mat individually to record their shape and weight on the GUI. Figure 9 and Figure 10 present the shapes and weights, respectively, obtained by placing the SKUs at various positions on the sensor mat. Based on the graph shown in Figure 6, the smallest weight that can be detected by each pixel of the system is 10 mN. Figure 9c shows the stacking items of two Nescafe metal cans (S9) and a heavy-weight SKU (S1) of 4.7 kg. The average percentage error derived for the weight of the SKUs was less than 4.3% and the highest error contribution was 10.8% for SKU S8 (Nescafe metal can, 258 g). It was observed that the bottom rim of SKU S8 has a minimum contact area with the sensing pixel elements of the sensor array. Hence, there is a need to reduce the dimensions of the sensing pixel to increase the accuracy of weight detection. Based on the experimental results, the system is demonstrated to be sensitive to weights of >250 g.

### 4.4. Automated SKU Identification Using Deep Learning

**Figure 11:**
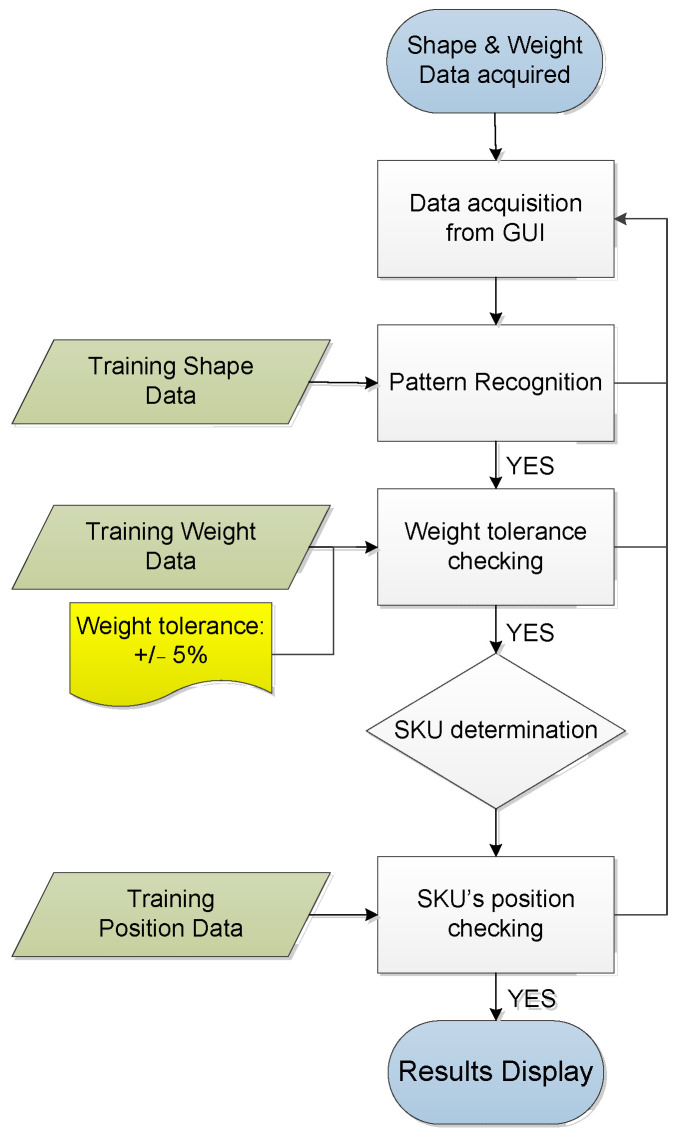
Logic flow chart of TensorFlow model.

In the system, a TensorFlow model is used for SKU identification through data provided by the sensor mat, which is connected to the GUI. Specifically, the shape and weight data of the SKUs, as acquired on the GUI, are used to identify SKUs placed on the sensor mat. The shape of a SKU plays a predominant role in SKU identification; hence, the model is trained by using multiple SKU features (e.g., bottom rim shape, rim diameter, and notch diameter). The addition of SKU weight features to the model further increased its automated detection accuracy. The TensorFlow algorithm compares the shape, weight, and position of the SKU with the training data as shown in Figure 11. The model is evaluated by using five SKUs as shown in Table 5; specifically, each SKU was placed on the sensor mat at various positions 20 times.

**Figure 12:**
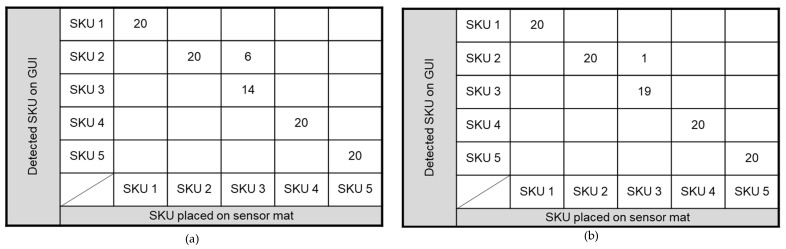
Confusion matrix of SKU identification using (**a**) only shape features and (**b**) both shape and weight features.

During model training, the SKUs are placed on the sensor mat such that their footprints were captured accurately; hence, the weight feature is also achieved. Figure 12b presents a confusion matrix that represents the performance of the model in identifying SKUs by using both shape and weight features, and Figure 12a presents a confusion matrix that represents the performance of the model in identifying SKUs by using only shape features. The SKU identification accuracy achieved using both shape and weight features was 99.2%, and that achieved using only shape features was 95%. As compared to the computer vision-based technology developed by Torrens et al. [10], the accuracy and spatial resolution of SKU detection by the developed interactive shelving system is 1.2% higher and four times smaller. Moreover, the developed system is cheaper than computer vision technology by 60%.

When only shape feature is used, it is observed that the identification errors are mainly associated with SKUs with similar bottom rim structures, such as SKU 3 (Coca-Cola; 520 g) and SKU 5 (Sprite; 354 g), which caused the identification accuracy to drop to 70%. However, incorporating both weight and shape features increased the identification accuracy to 95%. This finding implies that both shape and weight features should be included for SKU detection. Furthermore, the weight features of SKUs can be used to detect the stacking of SKUs. For example, if two Nescafe metal cans are stacked on top of each other, then it can use the weight feature to detect the presence of two cans. By contrast, the shape features of a SKU are insufficient for identifying SKU stacking. Thus, this provides a clear insight into the identification of a SKU using both shape and weight features. Hence, in the real-world scenario of SKUs with similar shape and weight there is a potential shortcoming of the current technology. Thereby, a camera-based scanner is needed as well to close the technology gap.

## 5. Conclusions

This study presents a novel interactive system for real-time inventory management in retail stores. The system comprises a sensor mat designed to facilitate stock-taking and misplaced item detection. This work has achieved the following results:

- A piezoresistive sensor mat is developed by fabricating a large sensor array of gold interdigitated electrodes covered with a carbon-based conductive polymer and a thin PET film. Each sensing pixel of the array has dimensions of 5 mm × 5 mm and an active sensing area of 4 mm × 4 mm.
- The sensor mat is characterized by using a benchtop force gauge, and the functional resistance range of the sensing pixels was determined to be between 14 and 65 kΩ. The minimal force for each sensing pixel was validated to be 10 g or 98 mN. A test of the repeatability of the sensor mat revealed an error of ±1.05%.
- The sensor mat’s durability was validated by dropping 1 kg standard weight.
- The developed sensor array was tested and validated by using eight SKUs, whose shapes and weights were displayed on a GUI. These SKU shape and weight features can be used in retail inventory management. The sensor array was determined to measure the weights of the SKUs with >95% accuracy.
- In the system, the GUI is integrated with a TensorFlow model. This trained model is assessed with five different SKUs and it was observed that when the model was trained using only SKU shape features, it achieved an identification accuracy of 95%. However, it achieved an identification accuracy of 99.2% when trained using both shape and weight features.

Usually, the footprint of SKU touching partially on the large-size sensing pixel may not activate due to insufficient force exerted on the sensing pixel. Decreasing the size of the pixels increases the coverage of the SKU’s bottom footprints on the sensing pixels, hence, activating large number of sensing pixels for detection. In the future work, the size of the sensing pixels will be reduced with the increase in the size of the sensor mat. This is to further increase its resolution of detection. In the real world, the size of the sensor mat must be altered to fit the shelves. Hence, we may need the integrated system of multiple sensor mats to be connected to the same circuit module. The modularized system is connected to the central PC through LAN connection. Hence, the overall implementation cost will be around USD 50,000, assuming that a retail store has 80,000 SKUs [33] and each sensor mat costing USD 12.5 can accommodate 20 SKUs. However, this set up involves large data consumption and demands high-speed LAN communication for mitigating the lag in the real-time stock management. In order to overcome this, it is recommended to use high frequency communication technology such as THz-based signal transfer [34].

In conclusion, a system comprising a piezoresistive conductive polymer-based sensor mat for real-time retail inventory management is demonstrated. The TensorFlow-based machine learning algorithm helps to improve the accuracy of the retail inventory system. Thus, the development of this system can help to promote industrialization and operational improvements. As the system does not collect any data from the customers or use any surveillance, it is claimed to be free of ethical or privacy considerations. Furthermore, it can reduce labor requirements and increase cost savings, thereby helping retailers to develop automated “humanless” retail stores.

## Figures and Tables

**Table 1:** Comparison of different technologies used for real-time inventory management.

Technology	Mechanism	Accuracy	Estimated Cost (US$)	Spatial Resolution	AI Embedded	References
EPC	Optical	95%	200,000 *	<20 mm	No	Inaba et al., 2019 [7]
RFID	Radio waves	93%	173,000 *	10 mm	No	Unhelkar et al., 2022 [8]
GPS	Trilateration	60%	200,000 *	5–10 m	No	Alodat et al., 2023 [9]
Computer vision	Image	98%	200,000	20 mm	Yes	Torrens et al., 2022 [10]
Piezo-capacitive sensors	Capacitive	73%	125,000	-	No	Lowe et al., 2004 [14]
Interactive Shelving System	Piezo-resistive	99.2%	50,000 ^#^	5 mm	Yes	This work

* Assuming the retail store has 10,000 items and including the manpower cost for tagging and implementation. ^#^ Assuming the retail store has 10,000 items and each sensor mat cost USD 100 and can accommodate 20 SKUs.

**Table 2:** Comparison of piezoresistive conductive polymers.

Product Name	Piezoresistive Conductive Polymer	Resistivity (ohms/sq/mil)	Remarks
Nano paint, Portugal [29]	InkPR02NP^®^	NA	Poor repeatability
Henkel, Germany [30]	Loctite ECI 7004Hr	17,300	Peeling-off in water
Adafruit Industries, New York [31]	Velostat Pressure- sensitive sheet	31,000	Thick form factor
MOS Corporation Ltd., Hong Kong [32]	AG125A	20,000	Repeatable, high temperature resistant and thin form factor

**Table 3:** SKU shape features experimental setup and heat map display result.

Conductive Polymer Thickness	SKUs on Sensor Mat	Display of SKUs on GUI	Remarks
6 μm	** 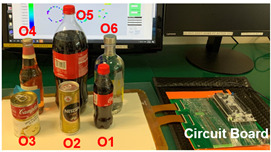 **	** 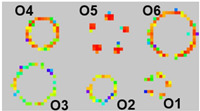 **	No missing Sensing pixels in O3
12 μm		** 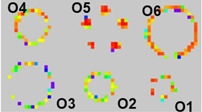 **	Missing sensing pixels in O3
37 μm		** 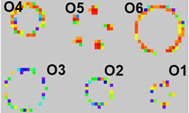 **	Missing sensing pixels in O3
O1: Coca-Cola Small, 250 mL O2: Nescafe metal can, 250 g		O3: Campbell metal can, 300 g O4: Somersby glass bottle, 330 mL	O5: Coca-Cola, 1.5 L O6: Vodka, 700 mL

**Table 4:** SKUs used for benchtop testing and validation.

SKU No.	SKU Name	Bottom Rim	Actual Weight (g)	Displayed Weight on GUI (g)
S1	Dish wash liquid	** 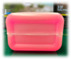 **	4760	4850
S2	pH balancer	** 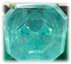 **	1552	1460
S3	Sprite small	** 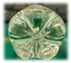 **	354	340
S4	Coca-Cola, 250 mL	** 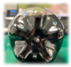 **	276	268
S5	Vodka, 700 mL	** 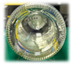 **	1166	1136
S6	White wine	** 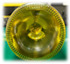 **	1200	1146
S7	Coke, 1.5 L	** 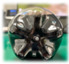 **	1556	1580
S8	Nescafe metal can	** 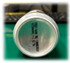 **	258	230

**Table 5:** SKUs used for training and automation detection using Tensor Flow model.

S. No.	SKU	Actual Weight (g)	Displayed Weight (g)
SKU 1	Vodka, 700 mL	1166	1136
SKU 2	Sprite small	354	340
SKU 3	Coca-Cola, 500 mL	520	498
SKU 4	Coke, 1.5 L	1556	1580
SKU 5	Nescafe metal can	258	230

## Data Availability

Not applicable.
